# Predictors of Short-Term Trauma Laparotomy Outcomes in an Integrated Military–Civilian Health System: A 23-Year Retrospective Cohort Study

**DOI:** 10.3390/jcm13071830

**Published:** 2024-03-22

**Authors:** Sami Gendler, Shaul Gelikas, Tomer Talmy, Roy Nadler, Avishai M. Tsur, Irina Radomislensky, Moran Bodas, Elon Glassberg, Ofer Almog, Avi Benov, Jacob Chen

**Affiliations:** 1 Israel Defense Forces, Medical Corps, Tel Hashomer, Ramat Gan 5262504, Israeljacopo669@gmail.com (J.C.); 2 Department of Medicine, Sheba Medical Center, Tel-Hashomer 5262504, Israel; 3 The National Center for Trauma & Emergency Medicine Research, Gertner Institute for Epidemiology and Health Policy Research, Sheba Medical Center, Ramat Gan 5262100, Israel; 4 Department of Emergency & Disaster Management, School of Public Health, Faculty of Medicine, Tel-Aviv University, Tel-Aviv-Yafo 6139001, Israel; 5 The Azrieli Faculty of Medicine, Bar-Ilan University, Safed 5290002, Israel; 6 The Uniformed Services, University of the Health Sciences, Bethesda, MD 20814, USA; 7 Department of Military Medicine, Faculty of Medicine, Hebrew University, Jerusalem 9112102, Israel; 8 Meir Medical Center, Kfar Saba 4428164, Israel; 9 Sackler Faculty of Medicine, Tel-Aviv University, Tel Aviv 69978, Israel

**Keywords:** trauma, laparotomy, military medicine, trauma systems, damage control resuscitation

## Abstract

**Background**: Trauma laparotomy (TL) remains a cornerstone of trauma care. We aimed to investigate prehospital measures associated with in-hospital mortality among casualties subsequently undergoing TLs in civilian hospitals. **Methods**: This retrospective cohort study cross-referenced the prehospital and hospitalization data of casualties treated by Israel Defense Forces-Medical Corps teams who later underwent TLs in civilian hospitals between 1997 and 2020. **Results**: Overall, we identified 217 casualties treated by IDF-MC teams that subsequently underwent a TL, with a mortality rate of 15.2% (33/217). The main mechanism of injury was documented as penetrating for 121/217 (55.8%). The median heart rate and blood pressure were within the normal limit for the entire cohort, with a low blood pressure predicting mortality (65 vs. 127, *p* < 0.001). In a multivariate analysis, prehospital endotracheal intubation (ETI), emergency department Glasgow coma scores of 3–8, and the need for a thoracotomy or bowel-related procedures were significantly associated with mortality (OR 6.8, *p* < 0.001, OR = 48.5, *p* < 0.001, and OR = 4.61, *p* = 0.002, respectively). **Conclusions**: Prehospital interventions introduced throughout the study period did not lead to an improvement in survival. Survival was negatively influenced by prehospital ETI, reinforcing previous observations of the potential deleterious effects of definitive airways on hemorrhaging trauma casualties. While a low blood pressure was a predictor of mortality, the median systolic blood pressure for even the sickest patients (ISS > 16) was within normal limits, highlighting the challenges in triage and risk stratification for trauma casualties.

## 1. Introduction

Hemorrhage remains the leading cause of preventable death in trauma patients, comprising up to 90% of military combat mortalities [1,2]. Despite advances in damage control resuscitation (DCR) and endovascular technology, trauma laparotomy (TL) remains the gold standard of emergency treatment for most trauma patients in profound hemorrhagic shock [3]. Therefore, early surgical capabilities are crucial in providing medical care to trauma victims, both in civilian and military scenarios [4].

In Israel, most military conflicts occur within close proximity to civilian populations, thus enabling Israel Defense Forces Medical Corps (IDF-MC) medical teams to provide prehospital care en route and rely on civilian medical centers for definitive care.

Considerable efforts have been invested into targeting specific indices, models, or scores that correlate with a casualty’s actual hemodynamic status and perhaps predict the need for various interventions, ranging from blood products to a TL [5]. Joseph et al. [4] evaluated casualty and interventional measures potentially affecting the survival of TL patients, finding that acidosis and coagulopathy were independently associated with TL mortality. However, data on prehospital interventions and measures associated with TL survival remain lacking.

This study aimed to characterize a cohort of casualties treated by IDF-MC military medical teams who underwent a TL in civilian hospitals. Our aim was to identify attributes that could assist in their triage, and we hypothesized that the implementation of innovative prehospital treatments in the field of damage control resuscitation would have a significant impact on patient outcomes.

## 2. Materials and Methods

### 2.1. Design and Study Population

This was a retrospective, registry-based cohort study.

This study included patients treated by IDF-MC teams in the prehospital setting who thereafter underwent a trauma laparotomy as documented in the Israel National Trauma Registry (INTR). All records from the IDF Trauma Registry (IDF-TR) from 1997 to 2020 were merged with their corresponding hospital data from the INTR using the patient’s identification number and date of injury. Israeli national identification numbers, which are unique and individualized, were used to assure proper cross-referencing. We excluded casualties with no record of identification number and casualties who were not admitted to hospitals included in the INTR, seeing as these could not be cross-referenced with certainty. Records that had an International Classification of Diseases-9 (ICD-9) procedure diagnosis of a laparotomy (or a sub-diagnosis of an exploratory laparotomy/other laparotomy 54.11, 54.12, 54.19) were included. The primary outcome of this study was in-hospital mortality.

The Israeli Defense Force Medical Corps (IDF-MC) Institutional Review Board approved the study (approval No. 2018-1948) and waived the requirement for written informed consent. The Helsinki Committee of the Sheba Medical Center approved the merger of the IDF-MC Trauma Registry data with hospitalization data from The Israeli National Trauma Registry (INTR) (approval No. SMC-14-1334). The manuscript was written and edited according to the STROBE statement guidelines [6].

### 2.2. Prehospital Trauma System

In Israel, prehospital care is provided by civilian as well as military teams. Israel’s main national emergency medical service is the Magen David Adom (MDA). IDF military medical teams, composed of paramedics and physicians, provide care for casualties in combat settings and care for civilian trauma that occurs in proximity to IDF bases and Israel’s borders.

Injured soldiers receive treatment in civilian hospitals because there are no military hospitals in Israel, except in times of war.

### 2.3. The IDF-MC Trauma Registry

The IDF-MC trauma registry is one of several military prehospital trauma registries in the world. It includes point-of-injury and prehospital data on military and non-military casualties treated by IDF-MC medical teams since 1997 [7]. The registry encompasses data on point-of-injury and en route care, relying on documentation on casualty cards and entries made by on-scene military medical providers to a web-based platform after the event.

### 2.4. The Israeli National Trauma Registry (INTR)

The INTR includes data from 21 trauma centers in Israel, including all level I trauma centers, accounting for approximately 95% of all hospitalized trauma cases. The registry indexes all patients hospitalized with an ICD-9 trauma diagnosis code of 800–959.9, including those pronounced dead in the emergency department (ED) or undergoing transfer to another hospital following injury. The INTR does not include casualties dying on-scene or during hospital evacuation, casualties discharged following treatment in the ED, or casualties admitted ≥72 h following the event. 

### 2.5. Variables

Demographic, point-of-injury, and prehospital data extracted from the IDF-TR included identification number, date of injury, sex, and age. Prehospital life-saving interventions (LSIs) included endotracheal intubation (ETI), cricothyroidotomy, tube thoracostomy, needle thoracostomy insertion and tourniquet application. Prehospital treatments included freeze-dried plasma (FDP), tranexamic acid (TXA) and crystalloid administration. Emergency department and hospital data extracted from the INTR included vital signs such as heart rate (HR) and systolic blood pressure (SBP) on arrival, Glasgow coma scale (GCS), airway status upon arrival, injury severity score (ISS), blood products transfusion, ICD-9 Clinical Modification (ICD-9-CM) codes for both diagnoses and procedures, hospital and ICU length of stay (LOS), and in-hospital mortality. ICD-9-CM codes were used to create the variables of exploratory laparotomy (54.11, 54.12, 54.19), as well as the additional surgical procedures these casualties underwent (bowel-related procedures, splenectomy, hepatobiliary procedures, thoracotomy, orthopedic, neurologic, and urologic procedures). 

### 2.6. Statistical Analysis

Categorical variables were compared using Chi-square and Fisher’s exact tests. Normally distributed quantitative variables were compared using Student’s *t*-tests. Other quantitative variables were compared using Mann–Whitney U tests. Categorical variables are presented as n (%) and continuous variables as means ± standard deviation (SD) or medians (interquartile range [IQR]) as appropriate. A comparison was made between casualty data according to ISS < 16 and ISS ≥ 16 as well as by survival. An ISS cutoff of ≥16 was used to discern between casualties undergoing laparotomies following major trauma and casualties with more minor trauma who underwent laparotomies. The ISS threshold of 16 has been commonly used to delineate major trauma [8,9]. Additionally, we compared casualties undergoing a laparotomy before and after 2014, seeing as the IDF implemented the use of TXA and FDP in late 2013 [10,11]. Univariate analysis and multivariate logistic regression were performed to assess the association of various factors with the primary outcome of in-hospital mortality. Factors included in the multivariate regression model, adjusted for ISS ≥ 25 and intraoperative hemorrhage control, were pre-selected according to the clinical basis as factors potentially affecting survival in laparotomy. These included the presence of a penetrating injury, the category of surgical operation, GCS 3–8, prehospital interventions, and the administration of blood products or ETI in the ED. Data processing and analyses were performed using R, version 4.0.3 (R Core Team, Vienna, Austria). *p*-values of <0.05 were considered significant.

## 3. Results

### 3.1. Patient Demographics and Injury Characteristics

Between 1997 and 2020, IDF-MC medical teams provided prehospital care for 11,118 casualties, of which 1361 (12.2%) died prior to hospital arrival. Among 4335 (38.9%) hospitalized casualties, 217 (5%) underwent a TL (Figure S1). No exclusions had to be made in the latter cohort, since all TL patients had proper identification numbers and their registry contained in-hospital data.

Table 1 summarizes the cohort’s injury characteristics and outcomes stratified according to injury severity by ISS. The main mechanism of injury was documented as penetrating for 121/217 (55.8%). The median SBP and HR in the ED were 124 mmHg and 100 BPM, respectively. Most casualties had a documented ED GCS of 15 (67.3%), while 26.4% had a GCS ≤ 8 and 6.2% had a GCS of 9–14. Of the total casualties, 74.2% had an ISS > 16 and 49.3% had an ISS > 25.

### 3.2. Prehospital Life-Saving Interventions

Documented prehospital LSIs included tourniquet application for 10 (4.6%) casualties, 24 (11.1%) tube or needle thoracostomy insertions, 23 (10.6%) endotracheal intubations, and 1 (0.5%) cricothyroidotomy. Prehospital treatments also included freeze-dried plasma (FDP) transfusions for 19 (8.8%) casualties, tranexamic acid for 26 (12%) casualties, and 50 patients (23%) received crystalloids.

### 3.3. Survival among Laparotomy Casualties

The overall in-hospital mortality of casualties undergoing an emergency laparotomy and included in the study was 15.2% (33/217). Most cases of mortality occurred within 24 h of hospitalization (25/33; 75.8%). Factors associated with mortality included arrival to the ED with ETI (54.5% in dead vs. 10.9% in surviving casualties; *p* < 0.001), head injury with AIS ≥ 3 (*p* = 0.019), GCS grouping (*p* < 0.001), ISS grouping (*p* < 0.001), and minimum SBP in the ED (*p* < 0.001). Notable factors not associated with differences in mortality were the maximum ED heart rate, mechanism of injury, and prehospital interventions including FDP administration, TXA administration, needle thoracostomy, tube thoracostomy and tourniquet application. The characteristics of the study population stratified according to mortality are depicted in Table 2.

### 3.4. ED and OR Interventions

Intra-operative procedures performed in the civilian hospitals are listed in Table S1. Forty-one (22.9%) casualties received blood products in the ED and fifty-one (23.5%) casualties underwent endotracheal intubation upon ED arrival. 

Bowel-related procedures were the most common, performed in 109 patients (50.2%); 37 (17.1%) underwent splenectomies and 21 (9.7%) underwent hepatobiliary procedures. In addition to TL, 37 (17.1%) casualties underwent orthopedic surgical procedures, 26 (12%) underwent a thoracotomy, and 13 (6%) underwent neurosurgical interventions. The median hospital LOS was 11 days. Regarding ICU stays, 136 (62.7%) casualties spent at least one day in the ICU and the median ICU LOS was one day (IQR 0–6.25; range 0–75).

In a univariate analysis for the primary outcome of in-hospital mortality (Table 3), including casualties with an ISS ≥ 16, we found that prehospital ETI was associated with mortality (OR 6.8, 95% CI 3–16.2). In addition, in the ED, a GCS of 3–8 and receiving a blood transfusion were associated with mortality (OR = 48.5, 95% CI 13.4–312.8 and OR = 4.61 95% CI 1.8–12.4, respectively). Intraoperative hemorrhage control and thoracotomy were also associated with mortality (OR = 4.2, 95% CI 1.8–9.8 and OR = 14, 95% CI 5.5–38.3, respectively). 

A multivariate model for in-hospital mortality adjusted for the ISS (16–24 and 25+) and intraoperative hemorrhage control was constructed. Prehospital endotracheal intubation (ETI), emergency department Glasgow coma scores of 3–8, and the need for a thoracotomy were significantly associated with mortality (OR 6.18, 95% CI 2.52–15.7, OR = 47.26 95% CI 12.3–319 and OR 14.4 95% CI 5.51–38.3, respectively) (Table 3).

### 3.5. Time Trends in Remote Control Damage Resuscitation (RDCR) Measures among Casualties Undergoing TL

Table S2 displays the characteristics of the study population split between two time periods, 1997–2013 and 2013–2020, to reflect changes in RDCR policies. Notably, FDP was administered to 19 casualties and TXA to 25 casualties in the second time period, with 1 casualty receiving TXA in 2013. We did not find significant differences in mortality between the two time periods (1997–2013: 14.8%, 2013–2020: 16.0%; *p* = 0.813).

## 4. Discussion

Patients who undergo a TL represent a subgroup of critically ill trauma patients in need of rapid evacuation to a medical facility. This is the first study conducted by the IDF-MC to evaluate outcomes following a TL. This study depicts a unique cohort, looking into a merged military–civilian database containing more than 20 years of prehospital and in-hospital treatment data. Moreover, this cohort represents a chimeric medical system where military casualties are treated at the point of injury by IDF-MC medical teams who treat them according to military clinical practice guidelines and are then transferred and operated on in modern civilian trauma centers by civilian surgeons. As the previous literature has demonstrated, there exists a dual relationship between military and civilian experience in trauma care, ultimately benefiting the patient [12,13]. Notably, we did not find that prehospital interventions, including FDP, TXA and application of needle thoracostomy or tube thoracostomy, were associated with differences in mortality among casualties undergoing a laparotomy. ED arrival following intubation or intubation in the ED was significantly associated with in-hospital mortality.

**Figure 1:**
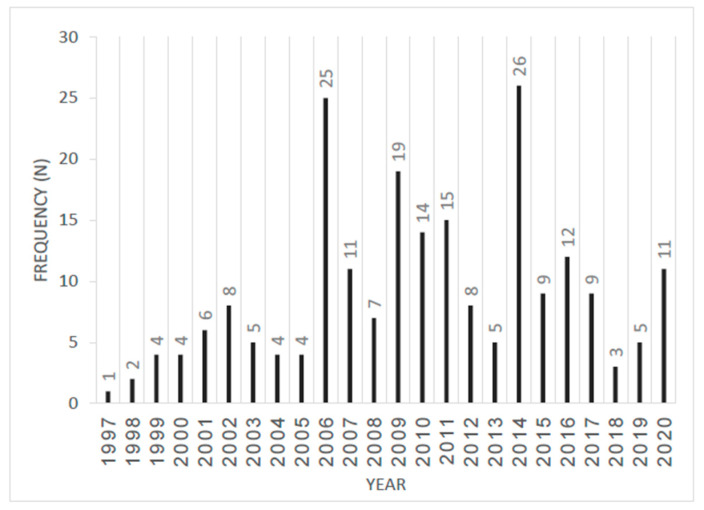
Distribution of trauma laparotomies throughout the study period (1997–2020) (N = 217).

The distribution of TLs over the years is consistent with Israel’s last three major conflicts (Figure 1): The Second Lebanon War (2006) and operations Cast Lead (2009) and Protective Edge (2014). Interestingly, it should be noted that the injury distribution between these conflicts did not differ significantly, with injury to the extremities being the most common followed by head, face, and then torso [14]. As the distribution of injuries has remained consistent throughout the study period, this finding can serve as guidance for the prospective initiatives of military health systems. Specifically, it can inform and direct future endeavors in the realms of injury prevention, the formulation of clinical practice guidelines, and the advancement of treatment modalities. Consequently, the in-hospital mortality observed in this cohort was 15.2%, which correlates with the high injury severity burden [15].

The overall in-hospital mortality rate (15.2%) described in our cohort mirrors that of a study by Marsden et al. [11]. In addition, Smith et al. [16] reported a 16% mortality in soldiers who underwent TLs in military-deployed role 2 or role 3 facilities. Simmons et al. also reported a 20% mortality rate among severely injured patients requiring both massive transfusions and exploratory laparotomies [17]. Muhrbeck et al. reported a notably low mortality rate among patients undergoing laparotomies in a tertiary civilian hospital in Iraq during the Battle of Mosul. However, as the authors stated, this observed low mortality was likely influenced by a significant survival bias, obscuring a potentially high prehospital mortality rate [18]. 

Over the 23-year span of this study, prehospital damage control resuscitation strategies in the IDF changed: Until 2003, the IDF endorsed the use of crystalloids and colloids to achieve “normal” vitals followed by a paradigm shift towards hypotensive resuscitation with small boluses of 250 mL of crystalloids. In 2010, the protocol changed again to a more balanced approach using 500 mL crystalloid bolus to achieve a systolic blood pressure above 80 mmhg [10]. In May 2011, following the CRASH-2 trial [19], tranexamic acid (TXA) was introduced as an adjunct to the prehospital resuscitation, which then was termed remote damage control resuscitation (RDCR), and in late 2013, freeze-dried plasma was also introduced to point-of-injury care (10). This evolution, alongside with the adoption of the combat application tourniquet (CAT, Composite Resources, Rock Hill, SC, USA) and its increased and aggressive use, led a reduction in battlefield mortality for the IDF [20]. This is evidenced by the low case fatality rate witnessed in operation “Protective Edge” as compared with previous conflicts [21]. Similarly, US military data have demonstrated that increased use of tourniquets and blood transfusions and more rapid prehospital transport were associated with a 44.2% decrease in total mortality [22]. Unfortunately, despite these changes, in our cohort of casualties undergoing TLs, these improvements in RDCR have yet to translate into a significant reduction in mortality, perhaps owing to the low number of casualties who underwent TLs since 2014 and the fact that mortality is a rare outcome. Moreover, considering the observed mortality benefit in the CRASH-2 trial (a 1.5% benefit to all-cause mortality and a 0.8% risk reduction in death due to bleeding) [19], a much larger sample size will be needed to evaluate for significance. Finally, it is reasonable to assume that due to the separate nature of the two systems (a military prehospital system transferring care to the civilian system), advancements made in one organization do not immediately translate to outcome improvements on the other side and a latency period is witnessed.

Predictive indices for mortality in trauma casualties are constantly sought. Traditionally, HR, SBP, and their ratio (represented in the shock index score) are commonly used to evaluate hemorrhagic shock [23,24]. In the current study, despite significant differences in HR and SBP among casualties with an ISS ≥ 16, both groups had a median HR and SBPs within the normal ranges. Moreover, HR and blood pressure were also similar and within the normal ranges regardless of the surgical procedure required, thus highlighting the challenges in triage and risk stratification for trauma casualties. It should be noted that while a lower minimum SBP was associated with in-hospital mortality, a maximal HR in the ED did not demonstrate such an association.

A higher mortality was also found among casualties who underwent prehospital endotracheal intubation. This finding is in accordance with the current global literature, as well as the IDF-MC’s experience with prehospital intubation, which showed that casualties who undergo prehospital intubation do not have improved outcomes [25,26]. In addition, prehospital intubation among casualties with hemorrhagic shock has been associated with increased in-hospital mortality [27]. Chao et al. identified prehospital intubation as an independent predictor of mortality in a civilian cohort [28]. It should be noted that in the adjusted model, severe head injuries (AIS ≥ 3) and a low GCS grouping (3–8) were also associated with mortality. Nevertheless, our data support limiting the use of prehospital intubation and reserving it for limited and specific scenarios where more conservative airway handling measures are insufficient. Hudson et al. [29] elaborated on the harmful effects of rapid sequence induction (RSI) intubation and positive-pressure ventilation (PPV) on patients in hemorrhagic shock, such as a reduced cardiac output, hypoxia, apnea, and a prolonged scene time. They concluded that intubating a bleeding casualty is “treating a “C” (circulatory) problem with an “A” (airway) solution”.

Exsanguination following trauma accounts for 90% of potentially survivable military deaths and 30–40% of trauma deaths [2]. This study suggests a higher mortality among casualties requiring intraoperative hemorrhage control and thoracotomy. Extremity tourniquets have been successfully implemented in military and civilian prehospital care (20). Prehospital control of bleeding from the torso and junctional areas remains challenging but offers a potential target for improving survival rates. There is still a great need to develop novel therapies to slow or stop non-compressible torso hemorrhages at the point of injury or en route to definitive care; however, overall, the utility of these retrievable adjuncts remains uncertain [30,31].

### Limitations

This study is limited by its retrospective design. Some data were unavailable or missing and point-of-injury medical care has changed throughout the study. Evacuation times were largely missing and were not included in the analysis. Finally, though applicable to military settings, the study is further limited by its population of mostly young, male, soldier casualties, which requires caution when generalizing the conclusions. 

## 5. Conclusions

Novel treatments introduced throughout this study period in the field of remote damage control resuscitation did not significantly affect in-hospital mortality among trauma patients undergoing emergency laparotomy. The severity of this cohort’s injuries, as reflected by the high ISS, did not translate to significant differences in maximal heart rates when comparing casualties according to survival, thus emphasizing the challenge of triaging these critically ill patients in need of emergency surgery. Finally, prehospital ETI was associated with in-hospital mortality. This finding reinforces previous observations suggesting that definitive prehospital airway interventions can have deleterious effects on trauma casualties.

## Figures and Tables

**Table 1:** Patient demographics, injury characteristics, and outcomes.

Characteristic	ISS 1–14 (N = 56)	ISS ≥ 16 (N = 161)	*p*-Value
Male sex, n (%)	52 (92.9%)	147 (91.3%)	0.717
Age, Median (IQR)	20 (19, 22)	20 (19, 25)	0.132
Patient population			0.303
Military	43 (76.8%)	112 (69.6%)	
Civilian	13 (23.2%)	49 (30.4%)	
Injury type			0.006
Penetrating	40 (71.4%)	81 (50.3%)	
Non-penetrating	16 (28.6%)	80 (49.7%)	
Severely injured region (AIS ≥ 3) *			
Head	0	34 (21.1%)	<0.001
Face	0	2 (1.2%)	0.4
Neck	0	3 (1.9%)	0.3
Thorax	5 (8.9%)	92 (57.1%)	<0.001
Abdomen	25 (44.6%)	137 (85.1%)	<0.001
Spine	1 (1.8%)	13 (8.1%)	0.09
Upper extremities	1 (1.8%)	18 (11.2%)	0.032
Lower extremities	4 (7.1%)	50 (31.1%)	<0.001
ED Vital Signs			
Max HR, BPM—Median (IQR)	90 (75, 102)	107 (88, 124)	<0.001
Min SBP, mmHg—Median (IQR)	130 (117, 145)	118 (92, 137)	0.003
Glasgow coma score			
3–8	1 (1.8%)	54 (35.3%)	<0.001
9–14	1 (1.8%)	12 (7.8%)	
15	53 (96.4%)	87 (56.9%)	
Injury severity score			
1–8	20 (35.7%)	-	
9–14	36 (64.3%)	-	
16–24	-	54 (33.5%)	
≥25	-	107 (66.5%)	
Arrived at ED with ETI	1 (1.8%)	37 (23%)	<0.001
Median hospitalization days (IQR)	6 (5, 11)	16 (6, 31)	<0.001
Intensive care unit admission	10 (17.9%)	126 (78.3%)	<0.001
Death	0	33 (20.5%)	<0.001

SD—standard deviation, IQR—interquartile range, ETI—endotracheal intubation, AIS—Abbreviated Injury Scale, ED—emergency department, HR—heart rate, BPM—beats per minute, SBP—systolic blood pressure, ISS—injury severity score. * Injuries could be sustained on more than one body region.

**Table 2:** Patient demographics, injury characteristics, prehospital interventions, and outcomes among casualties with a laparotomy stratified according to survival.

Characteristic	Survived after Laparotomy	Laparotomy Followed by In-Hospital Death	*p*-Value
	(N = 184)	(N = 33)	
Sex, Male, n (%)	170 (92.4%)	29 (87.9%)	0.489
Age, Median (IQR)	20 (19, 24.25)	20 (20, 23)	0.72
Patient population			0.404
Military	129 (70.1%)	26 (77.1%)	
Civilian	55 (29.9%)	7 (22.9%)	
Injury mechanism, Penetrating, n (%)	100 (54.3%)	21 (63.6%)	0.348
Prehospital LSI			
FDP	15 (8.2%)	4 (12.1%)	0.501
TXA	19 (10.3%)	7 (21.2%)	0.085
Needle thoracostomy	9 (4.9%)	4 (12.1%)	0.133
Chest drain	9 (4.9%)	2 (6.1%)	0.676
Tourniquet	8 (4.3%)	2 (6.1%)	0.651
Severely injured region (AIS ≥ 3)			
Head	24 (13.0%)	10 (30.3%)	0.019
Face	1 (0.5%)	1 (3.0%)	0.282
Neck	3 (1.6%)	0 (0%)	1
Thorax	78 (42.4%)	19 (57.6%)	0.129
Abdomen	135 (73.4%)	27 (81.8%)	0.388
Spine	12 (6.5%)	2 (6.1%)	1
Upper Extremities	15 (8.2%)	4 (12.1%)	0.501
Lower Extremities	43 (23.4%)	11 (33.3%)	0.274
ED Vital Signs			
Max HR, BPM, Median (IQR)	100 (84, 119)	110 (56, 130)	0.994
Min SBP, mmHg, Median (IQR)	127 (106.5, 140)	65 (56, 102.25)	<0.001
Glasgow coma score			<0.001
3–8	28 (15.6%)	27 (93.1%)	
9–14	12 (6.7%)	1 (3.4%)	
15	139 (77.7%)	1 (3.4%)	
ISS			<0.001
1–8	20 (10.9%)	0 (0%)	
9–14	36 (19.6%)	0 (0%)	
16–24	48 (26.1%)	6 (18.2%)	
≥25	80 (43.5%)	27 (81.8%)	
Arrived at ED with ETI	20 (10.9%)	18 (54.5%)	<0.001
24 h mortality	-	25 (75.8%)	
Mortality > 24 h	-	8 (24.2%)	

SD—standard deviation, IQR—interquartile range, LSI—life-saving intervention, FDP—freeze-dried plasma, TXA—tranexamic acid, ETI—endotracheal intubation, AIS—Abbreviated Injury Scale, ED—emergency department, HR—heart rate, BPM—beats per minute, SBP—systolic blood pressure, ISS—injury severity score.

**Table 3:** Univariate and adjusted analysis of the association of various factors with mortality.

Variable	Unadjusted			Adjusted *		
	OR	95% CI	*p*-Value	OR	95% CI	*p*-Value
ISS ≥ 25	2.98	1.22–8.44	0.025	-	-	-
Penetrating injury	1.98	0.91–4.48	0.089	1.58	0.65–3.88	0.3
Operation						
Bowel-related	0.43	0.19–0.94	0.03	0.42	0.18–0.93	0.042
Splenectomy	0.30	0.07–0.92	0.06	0.3	0.06–0.91	0.054
Hemorrhage control	4.25	1.85–9.81	<0.001	-	-	-
Thoracotomy	14.05	5.52–38.32	<0.001	14.4	5.51–38.3	<0.001
Neurosurgery	2.68	0.76–8.67	0.10	2.58	0.66–9	0.156
GCS 3–8	48.50	13.40–312.80	<0.001	47.26	12.3–319	<0.001
Prehospital treatments						
Freeze-dried plasma	1.47	0.39–4.65	0.53	1.65	0.4–5.78	0.45
Tranexamic acid	2.60	0.89–7.14	0.06	2.94	0.89–9.3	0.068
ETI attempt	4.20	1.60–10.94	0.003	4.25	1.51–12	0.006
Arrived at ED with ETI	6.88	3–16.26	<0.001	6.18	2.52–15.7	<0.001
In-hospital treatments						
Blood product in ED	4.61	1.79–12.38	0.002	2.07	0.83–5.21	0.115
ETI in ED	1.52	0.66–3.38	0.311	4.16	1.56–11.4	0.005

* Adjusted for ISS > 25 and intraoperative hemorrhage control. ETI—endotracheal intubation, ED—emergency department, GCS—Glasgow coma scale, ISS—injury severity score, CI—confidence interval.

## Data Availability

Data pertaining to individuals cannot be disclosed to ensure subjects’ anonymity and data security policies in the IDF. Additional data are available in the Supplementary Materials.

## Supplementary Materials

The following supporting information can be downloaded at: https://www.mdpi.com/article/10.3390/jcm13071830/s1, Figure S1: Flow diagram of the study cohort; Table S1: In-hospital and intraoperative procedures, Table S2: Casualty characteristics stratified according to time period.
