# Supplementary material for: Predictors of Short-Term Trauma Laparotomy Outcomes in an Integrated Military–Civilian Health System: A 23-Year Retrospective Cohort Study
Source: J Clin Med. 2024 Mar 22;13(7):1830. doi: 10.3390/jcm13071830 (PMC11012665; doi:10.3390/jcm13071830)
Supplement: Supplementary file 1 [file jcm-13-01830-s001.zip › jcm-2837165-supplementary.pdf]

Supplementary Materials

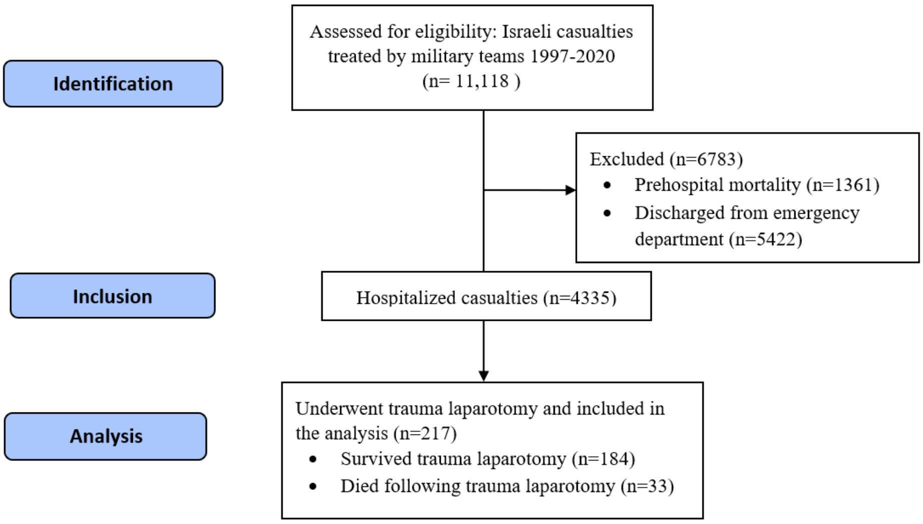

Figure S1. Flow diagram of the study cohort.

Table S1. In-hospital and intraoperative procedures.

Type of Procedure	ISS 1–14: Light and Moderate (N = 56)	ISS 16+: Severe and Critical (N = 161)	*p*-Value
Blood product transfusion in ED	2 (4.2%)	39 (29.8%)	<0.001
Endotracheal intubation in ED	4 (7.1%)	47 (29.2%)	<0.001
Operations			
Bowel-related	29 (51.8%)	80 (49.7%)	0.787
Splenectomy	2 (3.6%)	35 (21.7%)	0.002
Hemorrhage control	2 (3.6%)	36 (22.4%)	0.001
Procedure on diaphragm	1 (1.8%)	18 (11.2%)	0.032
Hepatobiliary	0	21 (13%)	0.004
Thoracotomy	0	26 (16.1%)	0.001
Orthopedic	2 (3.6%)	35 (21.7%)	0.001
Neurosurgery	0	13 (8.1%)	0.028
Urologic surgery	1 (1.8%)	15 (9.3%)	0.063
Eye procedure	1 (1.8%)	2 (1.2%)	0.764
Reoperation rate	3 (5.4%)	34 (21.1%)	0.007

Table S2. Casualty characteristics stratified according to time period.

Characteristic	1997–2013 (N = 142)	2014–2020 (N = 75)	*p*-Value
Sex			
Male	11 (7.7%)	7 (9.3%)	0.687
Female	131 (92.3%)	68 (90.7%)	
Casualty age			
Mean (SD)	22.1 (5.7)	26.7 (11.3)	<0.001
Median (IQR)	20 (19, 22)	21 (20, 30)	
Casualty population			
Military	109 (76.8%)	46 (61.3%)	0.017
Civilian	33 (23.2%)	29 (38.7%)	
Injury type			
Penetrating	78 (54.9%)	43 (57.3%)	0.735
Non-penetrating	64 (45.1%)	32 (42.7%)	
Prehospital LSI			
FDP	0 (0%)	19 (25.3%)	<0.001
TXA	1 (0.7%)	25 (33.3%)	<0.001
Needle thoracostomy	6 (4.2%)	7 (9.3%)	0.132
Chest drain	8 (5.6%)	3 (4.0%)	0.602
Tourniquet	3 (2.1%)	7 (9.3%)	0.016
ED vital signs			
Maximum HR, BPM			
Median (IQR)	103 (84, 120)	97.5 (83.75, 120)	0.347
Missing	6	7	
Minimum SBP, mmHg			0.688
Median (IQR)	123.5 (99.5, 140.25)	124 (101.5, 135.5)	
Glasgow coma score			
3–8	36 (26.5%)	19 (26.4%)	0.314
9–14	6 (4.4%)	7 (9.7%)	
15	94 (69.1%)	46 (63.9%)	
ISS			0.197

1–8	13 (9.2%)	7 (9.3%)	
9–14	26 (18.3%)	10 (13.3%)	
16–24	29 (20.4%)	25 (33.3%)	
≥25	74 (52.1%)	33 (44.0%)	
Mortality	21 (14.8%)	12 (16.0%)	0.813

SD—standard deviation, IQR—interquartile range, LSI—life-saving intervention, FDP—freeze-dried plasma TXA—tranexamic acid, ETI—endotracheal intubation, AIS—Abbreviated Injury Scale, ED—emergency department, HR—heart rate, BPM—beats per minute, SBP—systolic blood pressure, ISS—injury severity score.
